# Strong structural occupation ratio effect on mechanical properties of silicon carbide nanowires

**DOI:** 10.1038/s41598-020-67652-9

**Published:** 2020-07-09

**Authors:** Xuejiao Zhang, Jing Wang, Zhenyu Yang, Xuke Tang, Yonghai Yue

**Affiliations:** 1 School of Chemistry, Beihang University, Beijing, 100191 People’s Republic of China; 2 Institute of Solid Mechanics, Beihang University, Beijing, 100191 People’s Republic of China

**Keywords:** Materials science, Nanoscale materials, Nanowires

## Abstract

Materials’ mechanical properties highly depend on their internal structures. Designing novel structure is an effective route to improve materials’ performance. One-dimensional disordered (ODD) structure is a kind of particular structure in silicon carbide (SiC), which highly affects its mechanical properties. Herein, we show that SiC nanowires (NWs) containing ODD structure (with an occupation ratio of 32.6%) exhibit ultrahigh tensile strength and elastic strain, which are up to 13.7 GPa and 12% respectively, approaching the ideal theoretical limit. The ODD structural occupation ratio effect on mechanical properties of SiC NWs has been systematically studied and a saddle shaped tendency for the strength versus occupation ratio is firstly revealed. The strength increases with the increase of the ODD occupation ratio but decreases when the occupation ratio exceeds a critical value of ~ 32.6%, micro twins appear in the ODD region when the ODD segment increases and soften the ODD segment, finally results in a decrease of the strength.

## Introduction

Improving materials’ properties by designing novel structure is the most common method, therefore, it is vital to study the relationship between the structure and materials’ mechanical properties. Over the past two decades, SiC material has raised widespread concern due to its advantages such as large band gap, high thermal conductivity, high thermal stability, oxidation resistance, corrosion resistance and so on^1–4^. However, due to the lack of ductility at room temperature, its application has been dramatically limited. Recently, size effect is proved to be an effective strategy to improve materials’ properties^5–7^. For example, super-plasticity with local strain up to 200% in SiC nanowires (NWs) was observed^8,9^, similar phenomenon was revealed in other covalent system^10^. Besides the size effect, it is generally believed that materials’ mechanical properties critically depend on their internal structures at different length scales^11–13^. Both experimental and simulation results show that introduction of twin boundaries, grain boundaries and other internal structures can dramatically affect the mechanical properties of one-dimensional (1-D) materials^14–16^. For SiC NWs, they usually contain well-developed cubic (3C) structured segments, stacking faults, and also H-type structured segments and even helical structure^17^, dramatically affecting their mechanical properties, that may be the reason why the Young’s modulus of SiC NWs vary widely^18^. Recently, reserachers demonstrated that the stretchability of traditional brittle materials can be enhanced by buckling and channel guiding strategy^19–22^. Besides the structure mentioned above, one-dimentional disordered (ODD) structure with high density defects is another ubiquitous structure not only in SiC but also in other materials^8,23,24^, the symmetry and periodicity of the original face-centered cubic structure of SiC in the ODD structure change thoroughly^8^, which is considered to have not only great effect on mechanical properties but also on catalytic performance^24^. However, the mechanism how this structure affect the mechanical properties is still obscure. Herein, in situ tensile tests of single SiC NWs with different ODD occupation ratios (which are defined by the ODD structure volume ratios) have been conducted and a strong structure occupation ratio effect on mechanical properties is revealed for the first time. The strength increases with increase of ODD structural occupation ratio and then decreases when the occupation ratio approaches 36.2% with a maximum strength of 13.7 GPa, a saddle-shaped relationship strength versus ODD structural occupation ratio curve is demonstrated. Loading–unloading tensile tests show that the maximum elastic tensile strain of SiC NWs is ~ 12%, approaching its theoretical strain limit. Micro twins appear in the ODD region when the ODD segment increases and soften the ODD segment, finally results in a decrease of the strength. This work provides new insights into the structure effect on the mechanical properties of nanomaterials, and will be helpful for improving mechanical properties of new materials via structural design.

## Results and discussion

**Figure 1:**
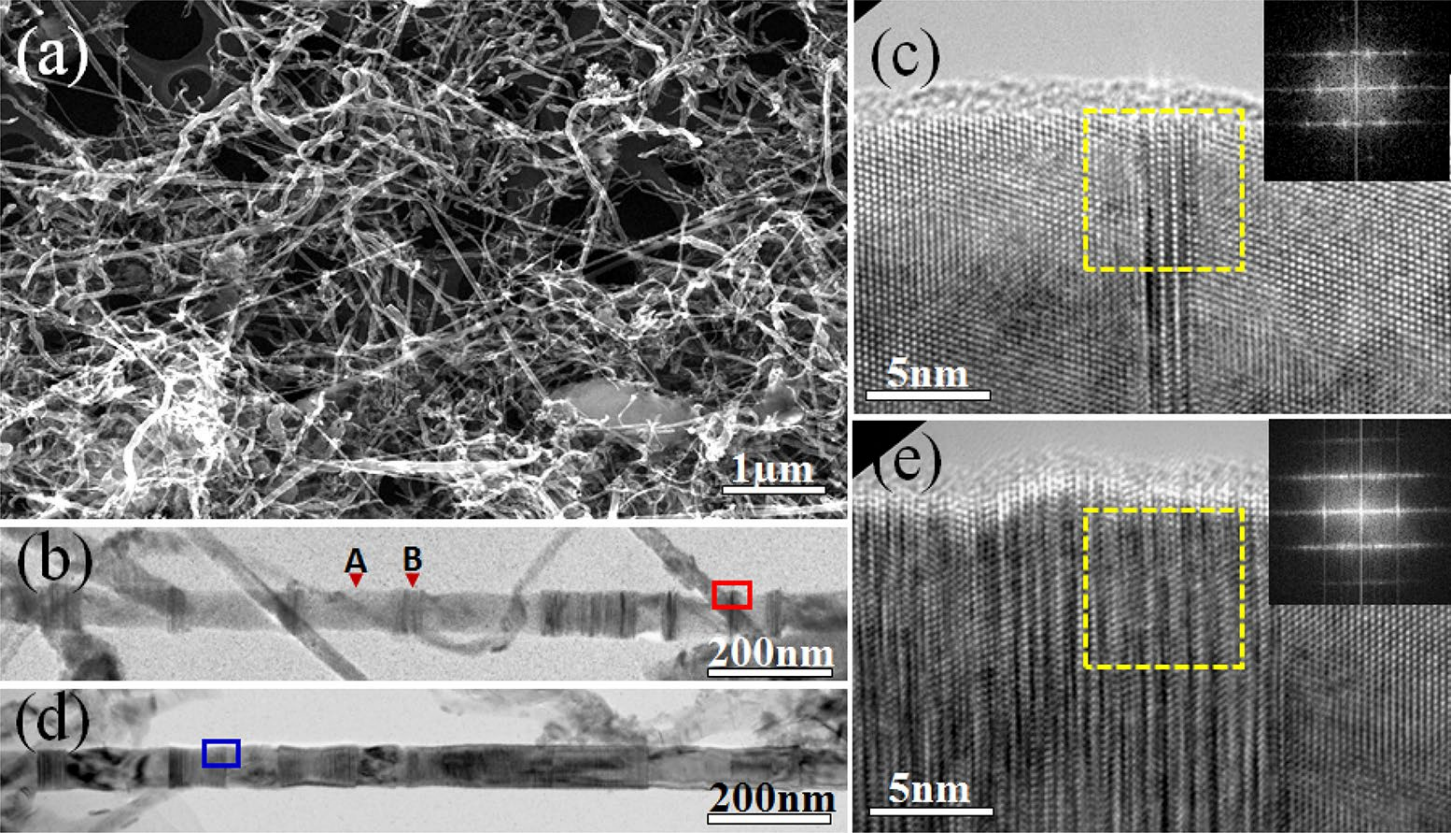
(**a**) SEM image of SiC NWs; (**b**) TEM image of a single SiC NW with a low ODD structural occupation ratio; (**c**) HREM image taken from the red framed region in (**b**); (**d**) TEM image of a single SiC NW with a high ODD structural occupation ratio; (**e**) HREM image taken from the blue framed region in (**d**).

High quality SiC NWs were prepared by the carbothermal reduction of the carbonaceous silicon xerogels containing lanthanum additive^25,26^. Figure 1a demonstrates the general morphology of the SiC NWs under scanning electron microscopy (SEM) observation. The average SiC NWs have a length of dozens of microns and with diameters ranging from several tens of nanometers to ~ 200 nm. Figure 1b shows a transmission electron microscopy (TEM) image of a single SiC NW consists of two type intergrowth segments as indicated by “A” and “B”, where segment “A” has smooth surface but segment “B” looks like a bamboo joint with rough surfaces^8^. With high resolution electron microscopy (HREM) image (Fig. 1c) taken from the red framed region in Fig. 1b, we find that segment “A” is the FCC structure, but segment “B” possesses ODD structure along 〈111〉 direction with stacking-faulted sequence of {111} plane which is along the longitude growth axis of the NW, insert in Fig. 1c shows the fast Fourier transform (FFT) image taken from the yellow framed region in Fig. 1c. Elongated spots in the insert image indicates the existence of the stacking-faults as demonstrated in Fig. 1c. Similar as Fig. 1b, d shows another single SiC NW but with a high ODD occupation ratio comparing with Fig. 1b. Figure 1e shows the HREM image taken from the blue framed region in Fig. 1d, and higher density ODD structure is further confirmed by the elongated spots which is nearly a line as shown in the FFT image (insert in Fig. 1e). In order to quantify the effect induced by the ODD segments, in situ tensile tests of single SiC NWs with different ODD structural occupation ratios have been conducted in SEM.

**Figure 2:**
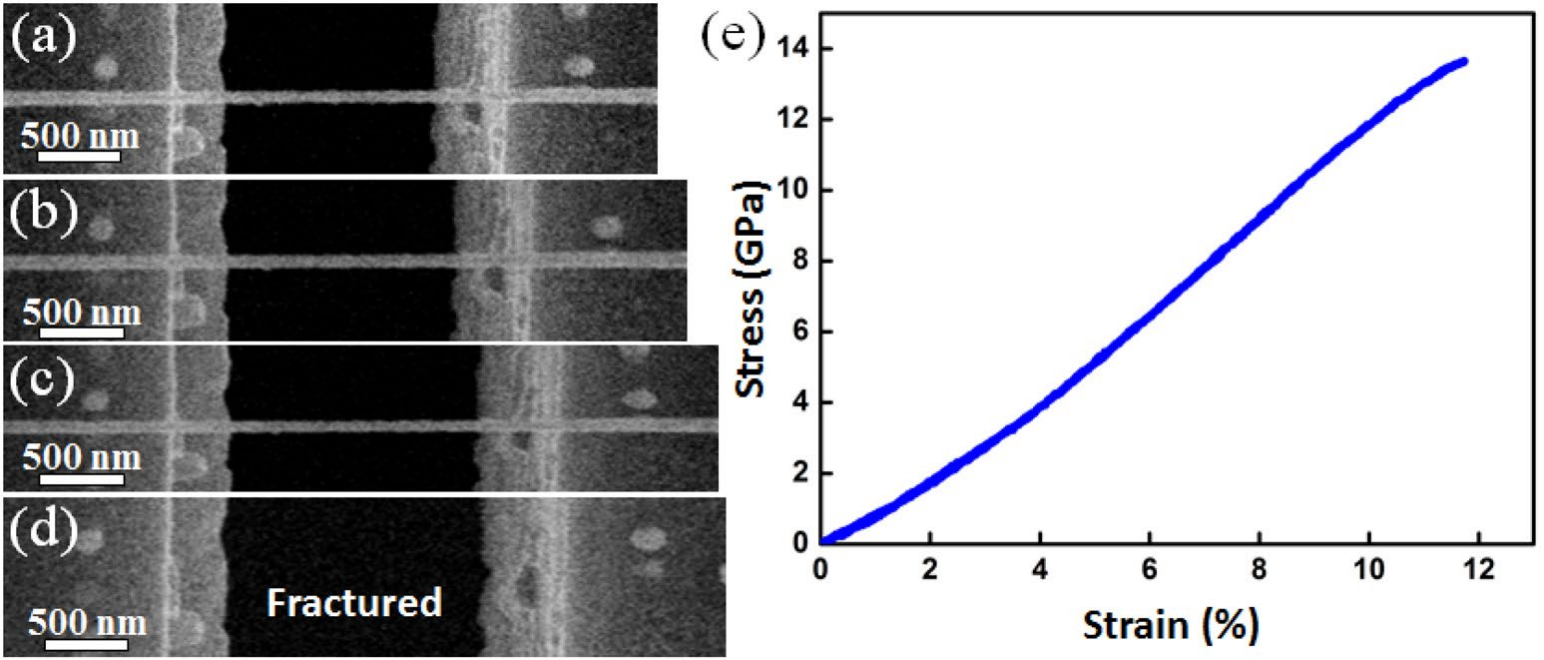
In situ tensile test. (**a**)–(**d**) A sequence of snapshots taken from Movie S1 showing the tensile process of a single SiC NW; (**e**) Stress–strain curve of the single SiC NW.

Uniaxial tensile tests of single SiC NWs were performed using an in situ quantitative nanoindenter (Hysitron Pi-85) inside a FEI Quanta 250 FEG SEM. A dual beam (focus ion beam, FIB-SEM) system was used to transfer and fix SiC NWs. A tungsten probe was used to pick up the target SiC nanowire and put it to the gap of the push-to-pull (PTP) device, then Pt patterns were deposited to the two ends of the SiC NW to fix it as shown in Figure S1a. The basic configuration of the tensile tests was also shown in Figure S1, after the 20 μm flat probe was positioned to touch the semi-circular end of the PTP device, the indentation force converted to tensile force and loaded to the yellow dashed line framed region as shown in Supplementary Fig. S1a. Uniaxial tensile force loaded (along the direction marked by the two yellow arrows) to the tested SiC NW that was fixed on the device by FIB. Meanwhile, the force and displacement curve and a real-time movie were recorded dynamically. The true force-versus-displacement curve of a tested sample could be accurately extracted and transferred to stress–strain curve by removing the contribution from the free PTP device (See Supplementary Fig. S2 online). Supplementary Fig. S1b displayed a single SiC NWs with lots of bamboo joints as denoted by the yellow arrow, which was fixed on a PTP device by FIB. Figure 2a–c demonstrated a sequence snapshots extracted from the Movie S1, which was captured during the tensile test of a SiC NW with an ODD structural occupation ratio of 32.6%, the maximum strain approached ~ 12%, while the calculated stress reached about 13.7 GPa as shown in the stress–strain curve in Fig. 2e, then, the SiC NW fractured and ejected without obvious plastic deformation as shown in Fig. 2d, e.

**Figure 3:**
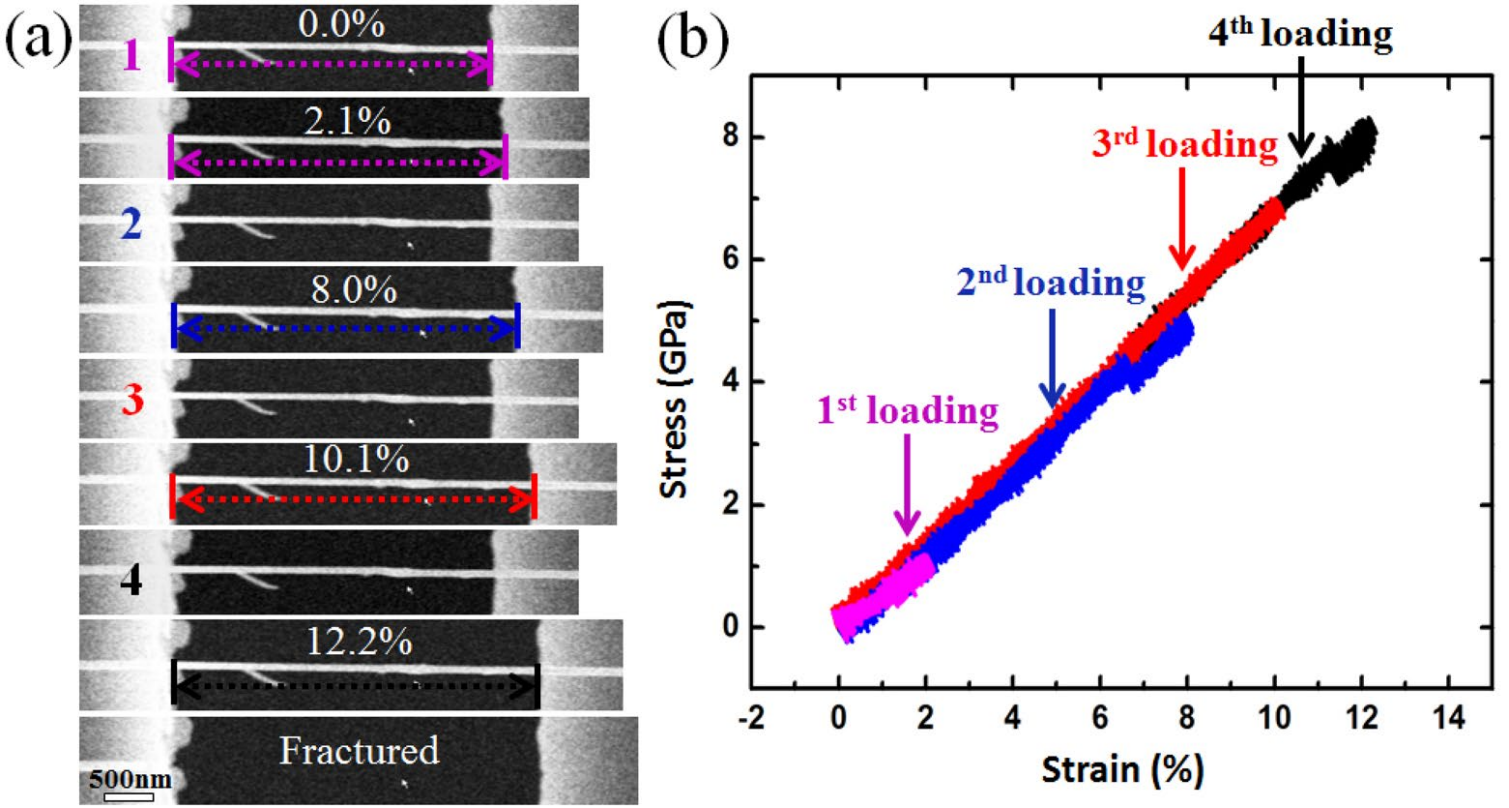
(**a**) Loading–unloading tensile test of a single SiC NW with increasing tensile strain amplitude and full unloading in each cycle. The nanowire recovered its original length after strain values of ~ 2.1, ~ 8.0, and ~ 10.1% were experienced in each cycle and eventually fractured at the fourth cycle with a final strain of ~ 12.2%, the broken nanowire flew away as shown in the last snapshot in (**a**). (**b**) Corresponding stress-versus-strain curves of the multicycle loading–fully unloading test, different colors were used to better illustrate the data for each cycle.

Loading–unloading experiments were performed under SEM to confirm that the SiC nanowire strain could be fully recovered after very large strains were experienced upon unloading. Four loading–unloading cycles were performed on a single SiC NW with an ODD structural occupation ratio of 20.1%, as shown in Fig. 3a, the loaded maximum strain increased gradually during these four cycles. The SiC NW finally fractured at a strain of ~ 12.2% with a fracture strength of ~ 8 GPa. As shown in Fig. 3b, all the stress–strain curves showed linear and hysteresis free shape during the loading–unloading cycles. More details could be found in Movie S2 in supporting materials. Unixal tensile tests of several SiC NWs were performed to verify the repeatability of the super large elastic strain and the measured maximum elastic strain of SiC NWs is ~ 15.5%, approaching the theoretical strain limit of semiconductor materials^27^. As reported in previous result^28^, all tested SiC NWs demonstrated a brittle-like fracture character which may mainly due to the relative high strain rate that is about 1.2 × 10^–3^ s^−1^, it is fast and there would be no enough time for phase transformation from crystal to amorphous which would result in a super plasticity^9^. Such high strain rate was too fast for the nanowire to experience a plastic process induced by bond switching and rebonding^29,30^.

**Figure 4:**
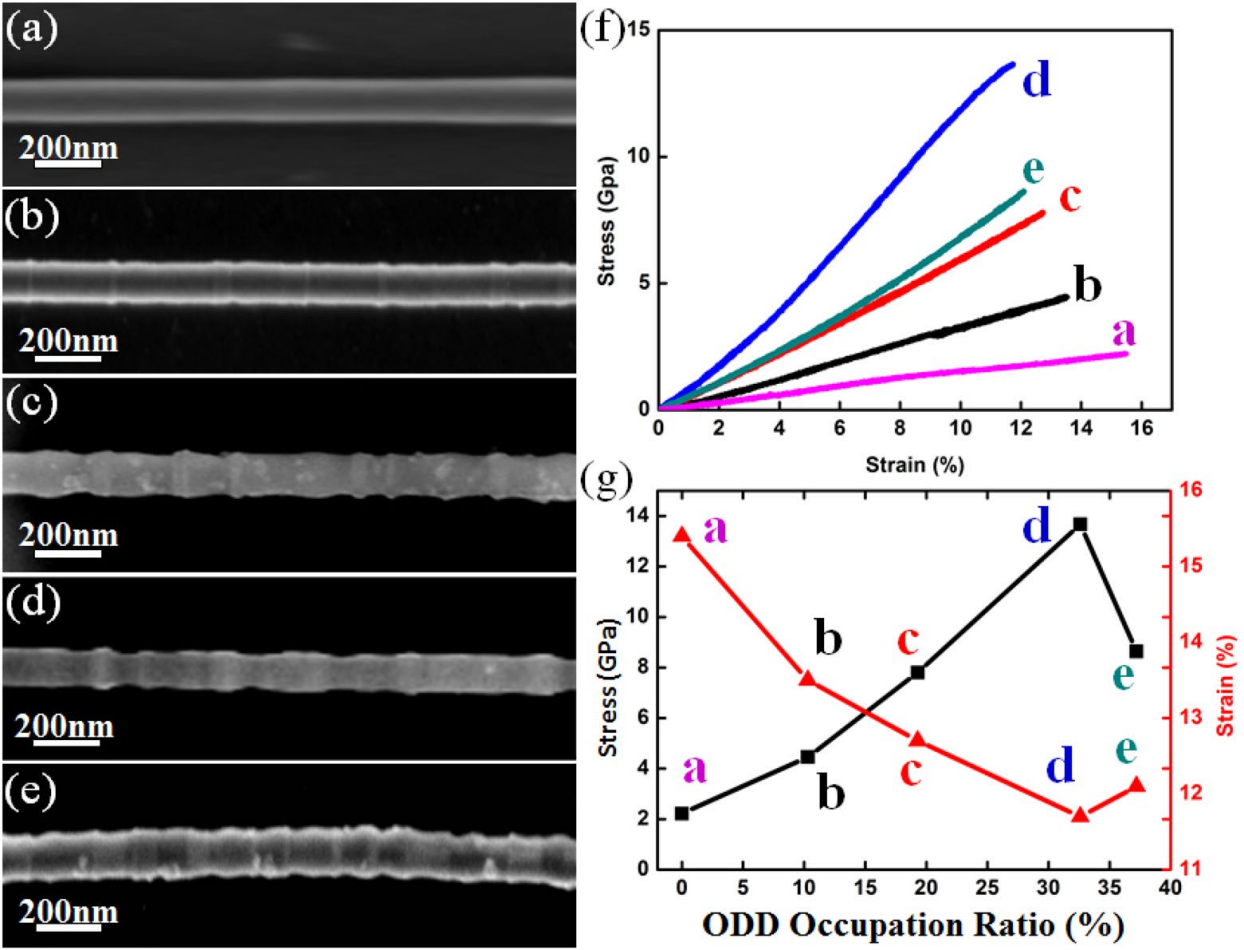
(**a**)–(**e**) SEM images showing different single SiC NWs with different structural occupation ratio of 0%, 10.3%, 19.3%, 32.6%, 37.2%. (**f**) corresponding stress–strain curves of SiC NWs demonstrated in (**a**)–(**e**); (**g**) stress/strain-ODD occupation ratio relationship.

During the tensile tests of SiC NWs, we found that the strength varies greatly with the change of the ODD occupation ratio. In order to map out the relationship between the ODD occupation ratio and the mechanical properties, SiC NWs with different ODD occupation ratios but with similar diameter have been picked up to conduct the tensile tests. Selecting nanowires with similar diameter aims to rule out the size effect on the mechanical properties^31^, then, the difference in mechanical properties is mainly due to structural changes. Figure 4a–e demonstrates 5 single SiC NWs with different ODD occupation ratios which are ranging from 0 to 37.2%. The occupation ratio was calculated according to the volume fraction of the ODD segments. All corresponding stress–strain curves of these SiC NWs are presented in Fig. 4f. Most of these stress–strain curves demonstrate good linearity. The strength increased with increasing of the ODD occupation ratio at the beginning but decreased when the occupation ratio was higher than 32.6%, and the highest strength was approaching ~ 13.7 GPa, near the therotical strength limit^27^. Curiously, the strength didn’t increase with further increase of the ODD structural occupation ratio after the critical value of 32.6%, it decreased when the ODD occupation ratio was higher than this value and a saddle like ODD occupation ratio effect on the strength was first demonstrated as shown in Fig. 4g. Different with the tendency strength, the strain demonstrated a antipodal tendency comparing with the strength, it decreased with increasing of the ODD structural occupation ratio and increased when the occupation ratio reached higher than 32.6% as shown by the red line in Fig. 4g.

**Figure 5:**
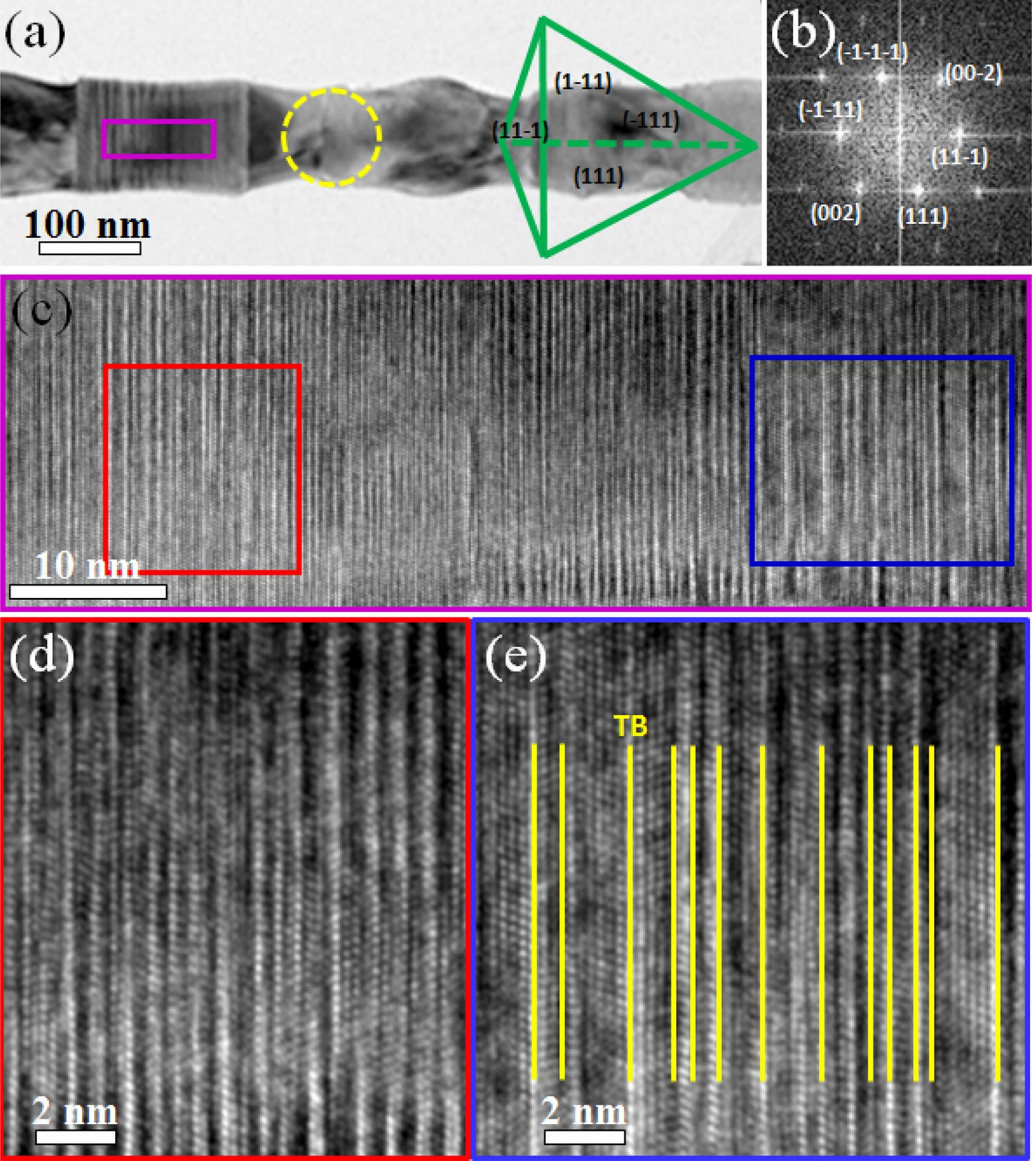
(**a**) TEM image of a single SiC NW with ODD structure occupation ratio of ~ 38%, Thompson tetrahedron showing the orientation relationship of the 4 {111} planes; (**b**) FFT image taken from the yellow dashed circle region in (**a**); (**c**) Enlarged HREM image taken from the pink framed region in (**a**); (**d**) and (**e**) are enlarged HREM images taken from the red and blue framed regions in (**c**), yellow lines in (**e**) display TBs.

For these two kinds of SiC segments, comparing with triditional 3C structure, ODD segment could be consided as a kind of hard phase while 3C structure was considered as the soft phase, as revealed in nacre or manmade nacre-like materials^32,33^, the ratio between hard phase and soft phase is esstional to the mechanical properties of composites^34,35^. When the hard phase proportion increased, the strength increased but would result in a decrease of the total strain as shown in Fig. 4g. But why the strength displays a saddle like tendency with the ODD occupation ratio? After we further studied the structure of the large ODD segment, we found the reason that also originated from the interior structure of the ODD. Figure 5a shows a TEM image of single SiC NW with an ODD occupation ratio of ~ 38%. High density ODD regions were demonstrated clearly. After we enlarged the pink framed region as shown in Fig. 5b, we found that besides the high density ODD structure (Fig. 5c), there were also many micro-twins existing within the ODD structure as shown in Fig. 5d, with the highest thickness approached ~ 2 nm. As mentioned in previous study^36^, angstrom-scaled twins (0.7 nm in thickness) could help materials approach its theoretical strength, but, homogeneous nucleation of dislocations inside the NWs followed by shear localization and confined microplasticity at twin boundaries (TBs) would occurred when the twin thickness increased, leading to an increase of strain but a decrease of the strength. Herein, the appearance of micro-twins in ODD structure finally decreased the strength of the ODD structure, the total strain of SiC NWs with a higher ODD occupation ratio had hit bottom and begun climbing again as shown in Fig. 4g, displaying a contrary tendency to the tendency of strength.

**Figure 6:**
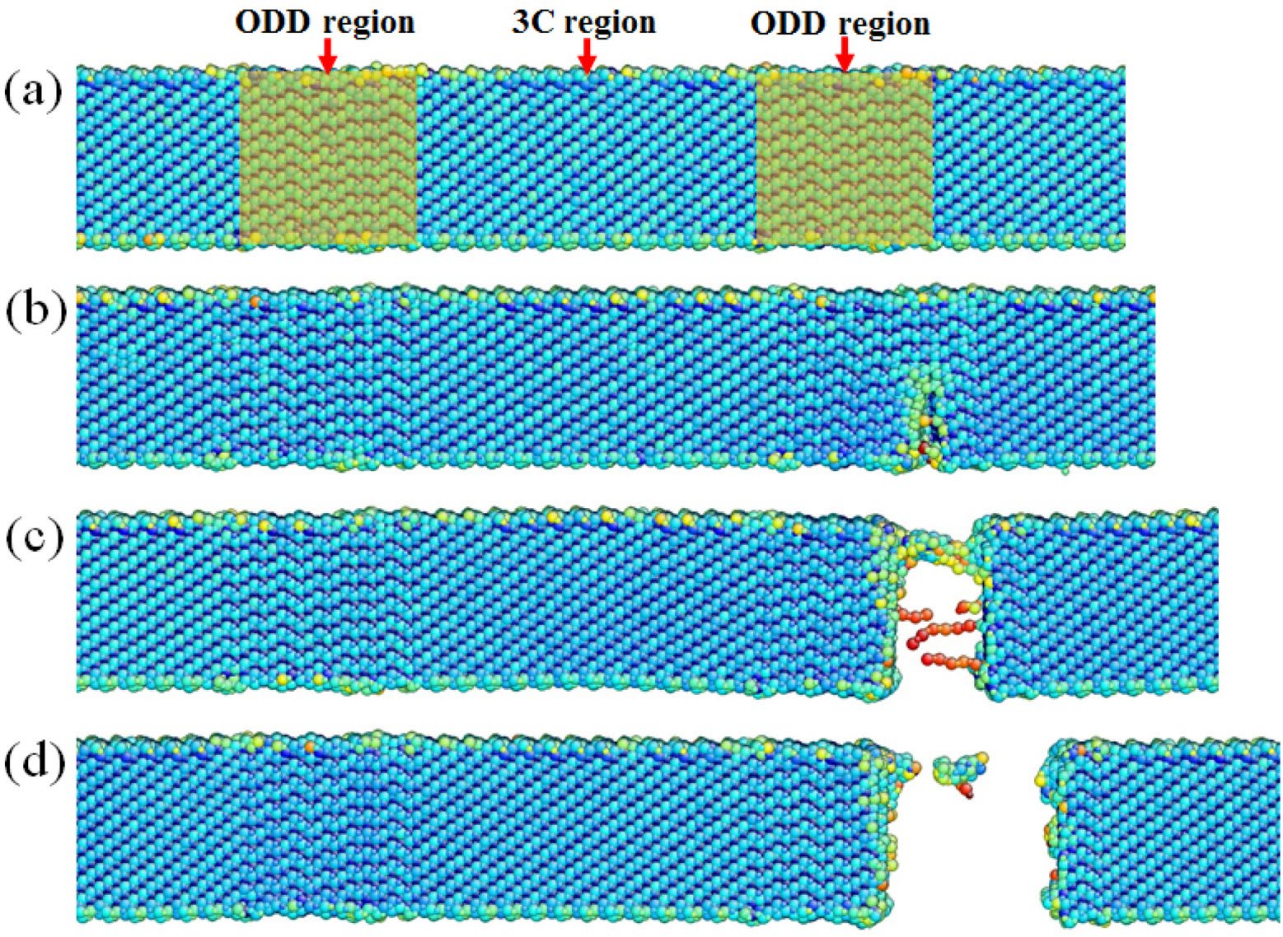
MD simulation result of the SiC nanowire with periodical ODD structure, the ODD occupation ratio is 33.3%. (**a**)–(**d**) show the extracted snapshots taken from the tensile process, the two orange shadow areas indicate the ODD regions.

In our tesile tests, all tested SiC NWs demonstrated a brittle-like fracture feature. Take Fig. 5a as an example, the force loading direction is along [11−1]. Stacking sequence in 3C-SiC segments was ABCABC…(A, B, C represents the three basic structure modules of tetrahedral bonding in SiC^37^), creating a straight atomic plane along all of the 4 {111} planes ((1−11), (11−1), (111) and (−111) plane (as demonstrated in Fig. 5b) which were translational symmetry and periodicity in these four {111} planes, and the green tetrahedron in Fig. 5a demonstrated the Thompson tetrahedron, showing the orientation relationship of these four {111} planes. However, stacking sequence in the ODD structure was in a random order, such as ACABABCB…, the translational symmetry and periodicity were broken in the three sets of (1−11), (111), and (−111) planes except for the (11−1) plane. When yielding happened, free surface in ODD structure possessed high energy and would act as dislocation source to emit dislocations, but all the three sets of (1−11), (111), and (−111) sliding planes were broken due to the discontinuous slipping plane, dislocation emission along these three planes was almost impossible. Furthermore, the force loading direction was vertical to (11−1) plane, and all types of dislocations on (11−1) plane including full dislocations along 〈110〉 and partial dislocations along 〈112〉 had a Schmid factor of zero which was impossible for dislocation movement in (11−1) plane, so dislocation behavior could be found only in 3C segments under a low strain rate as report before^9^. If the tensile test was conducted at a high strain rate, stress concentration was fast enough to form a crack on the ODD segment surface and brittle fracture would happen^28^ under such a high strain rate of 10^−3^ s^−1^. Such brittle fracture phenomenon shown in Fig. 2 and Supplementary Fig. S3 (See more details in Movie S3) were further confirmed by the MD simulation result as demonstrated in Fig. 6, a constant tensile force was loaded to the SiC NW with a strain rate of ~ 10^8^ s^−1^. The nanowire was constructed according to the experimental result with an ODD occupationa ratio of 33.3% to study the fracture mode. Figure 6a–d demonstrate a cleavage fracture model which was in accordance with our experimental results, small crack appeared near the ODD/3C interface region as shown in Fig. 6b, where no obvious dislocation behavior were found during the fracture process and the crack propagated rapidly to another side of the nanowire, resulting in a brittle failure as shown in Fig. 6d. In order to prove our speculation mentioned above, twin lamella were inserted into the ODD region and similar tensile test was conducted as shown in Supplementary Fig. S4. As predicted, fracture happened in the twin lamellae with the same fracture model as shown in Fig. 6 but with a slight decrease in strength (see Supplementary Fig. S4 online), in accord with our experimental result, further proved our interpretation of the strong ODD occupation effect on the strength of SiC NWs.

## Methods

### Characterization

We used a Quanta 250 FEG SEM to characterize the morphology of the SiC NWs. A JEOL 2100F TEM at an accelerate voltage of 200 kV was employed to study the structure of the SiC NWs.

### In situ tensile test

We conducted the in situ tensile tests of single SiC NW by a Pico Indenter (Pi-85 from Bruker) in a SEM at an accelerate voltage of 10 kV. FIB was employed to transfer and fix the single SiC NW onto the PTP device. We use the displacement control mode to pull the single SiC NW until its fracture at a displacement rate of 2 nm s^−1^. The force VS displacement curve accompanied by the real-time video were recorded dynamically.

### Quantitative analysis

SEM was employed to characterize the initial length and the diameter of the tested SiC NW. The stress and the strain were calculated based on the diameter and the elongation. Both the sample and the PTP device contributed to the force. Thus, we subtracted the force contributed by the PTP device to calculate the stress.

### MD simulation

The MD simulations were performed using the large-scale atomic molecular massively parallel simulator (LAMMPS), and the Vashishta potential was applied for the silicon and carbon atoms. ODD segments were simulated with a given stacking sequence. We applied a periodic boundary condition along the nanowire axis and relaxed the model for 50 ps with a NPT ensemble before the deformation. Then, we loaded the model in a NVT ensemble until its fracture.

## Conclusion

ODD structure is a kind of particular structure in SiC, which highly affects its mechanical properties. In this study, with in situ tensile tests of single SiC NWs with different ODD occupation ratios in a SEM, we demonstrated that SiC NW with an ODD occupation ratio of 32.6% exhibits an ultrahigh tensile strength and a super large elastic strain which are up to 13.7 GPa and 12%, respectively, near the ideal theoretical limit. A strong saddle shaped ODD occupation ratio effect on the mechanical properties was revealed for the first time. The strength increased with the increase of the ODD occupation ratio but decreased when the occupation ratio exceeded a critical value of ~ 32.6%, micro twins appeared in the ODD region when the ODD segment increased and softened the ODD segment, finally resulted in a decrease of the strength. This work may shed light on the strategy to modify materials’ mechanical properties by structure design.

## Supplementary information

Supplementary Information 1.

Supplementary Information 2.

Supplementary Information 3.

## Data Availability

All data generated or analyzed during this study are included in this published article (and its Supplementary Information files).
