# Supplementary material for: Strong structural occupation ratio effect on mechanical properties of silicon carbide nanowires
Source: Sci Rep. 2020 Jul 9;10:11386. doi: 10.1038/s41598-020-67652-9 (PMC7347842; doi:10.1038/s41598-020-67652-9)
Supplement: Supplementary file 1 — Supplementary Information 1. [file 41598_2020_67652_MOESM1_ESM.docx]

Supporting information for “Strong Structural Occupation Ratio Effect on Mechanical Properties of Silicon Carbide Nanowires”

Xuejiao Zhang, ^†#^ Jing Wang^†#^, Zhenyu Yang, ^‡#^ Xuke Tang^†^, Yonghai Yue^†^*

^†^School of Chemistry, Beihang University, Beijing 100191, P. R. China

^‡^Institute of Solid Mechanics, Beihang University, Beijing 100191, P. R. China

^#^These authors contributed equally to this work

E-mail: [yueyonghai@buaa.edu.cn](mailto:yueyonghai@buaa.edu.cn);

1. *In situ* tensile test

A FEI Quanta FEG 250 SEM and a JEOL 2100F TEM are used to characterize the SiC NWs. The *in situ* tensile tests are conducted by a quantitative nanoindenter (Hysitron Pi-85) with a particular PTP device. FIB is employed to transfer and fix the SiC NW. We use a tungsten probe to pick up the target SiC nanowire and put it to the gap of the push-to-pull (PTP) device. Pt patterns are deposited to the two ends of the SiC NW to fix it. The basic configuration of the tensile tests is shown in Fig. S1, after the 20 μm flat probe is positioned to touch the semi-circular end of the particular push-to-pull (PTP) device, the indentation force converts to tensile force and load to the yellow dashed line framed region in Supplementary Fig. S1a. Supplementary Fig. S1b shows the enlarged SEM image taken from the yellow framed dashed line region in Supplementary Fig. S1a, bamboo joints as marked by the yellow arrow in Supplementary Fig. S1b demonstrate the ODD segments. Double heads arrow in Supplementary Fig. S1b displays the force loading direction.


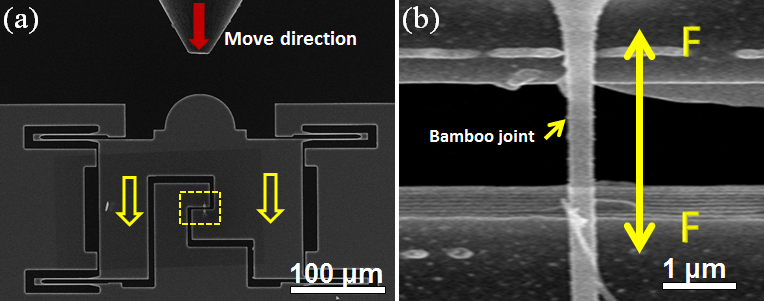


**Supplementary** **Figure S1.** (**a**) the basic configuration of a PTP device and (**b**) enlarged SEM image taken from the yellow framed region in (**a**).

Supplementary Fig. S2 shows the representative Load-Displacement curve of the SiC NW on the PTP device. When the displacement was at ~180 nm, the NW broke and the force signals were tested only for an empty PTP device, which showing obvious linear responds. The calculated elasticity modulus of the empty PTP device was 43.9 N/m. We would get the force and displacement curve of the NW, after subtracting the force of the PTP device.


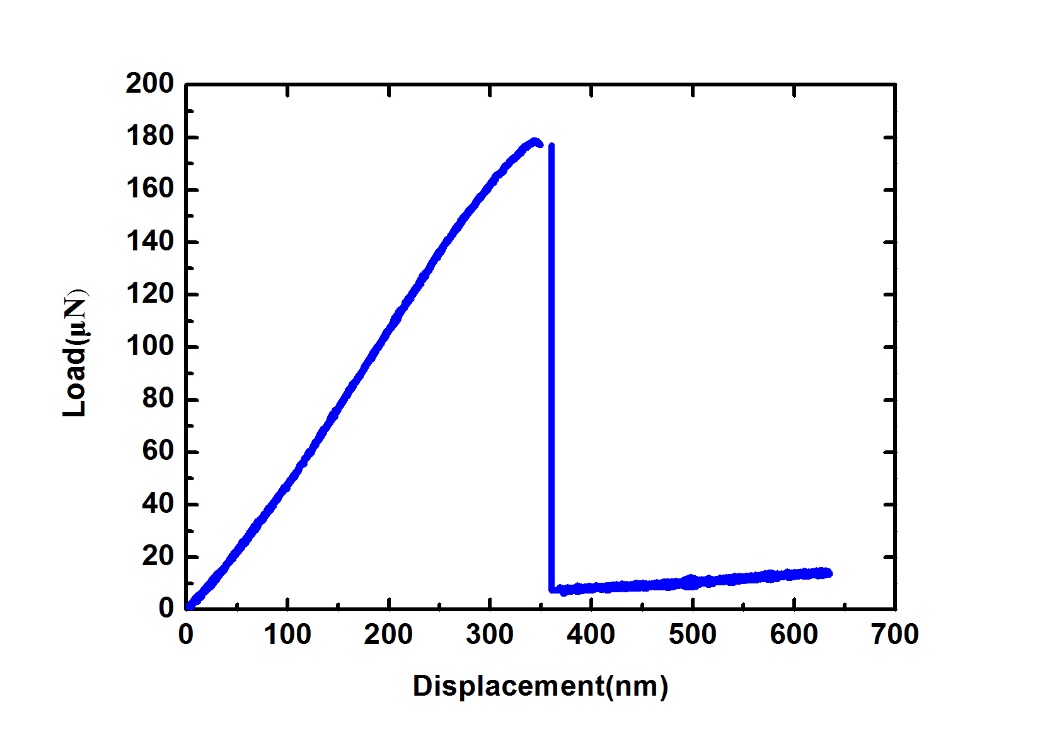


**Supplementary Figure S2**. Representative Load-Displacement curve of the SiC NW on the PTP device. The calculated elasticity modulus of the empty PTP device was 43.9 N/m. We would get the force and displacement curve of the NW, after subtracting the force of the PTP device.

1. Another example shows the brittle fracture feature of the SiC NW with an ODD occupation ratio of 37.2%.

Supplementary Fig. S3 shows another tensile test of the SiC NW with an ODD occupation ratio of 37.2%, Supplementary Fig. S3a-S3e show snapshots taken from the Movie S3. A brittle fracture feature was found as demonstrated by the yellow arrow in Supplementary Fig. S3e. Supplementary Fig. 3f shows the Force-Displacement including the effect of the empty PTP device as shown in Supplementary Fig. S2. Supplementary Fig. 3g shows the calculated stress-strain curve of this SiC NW.


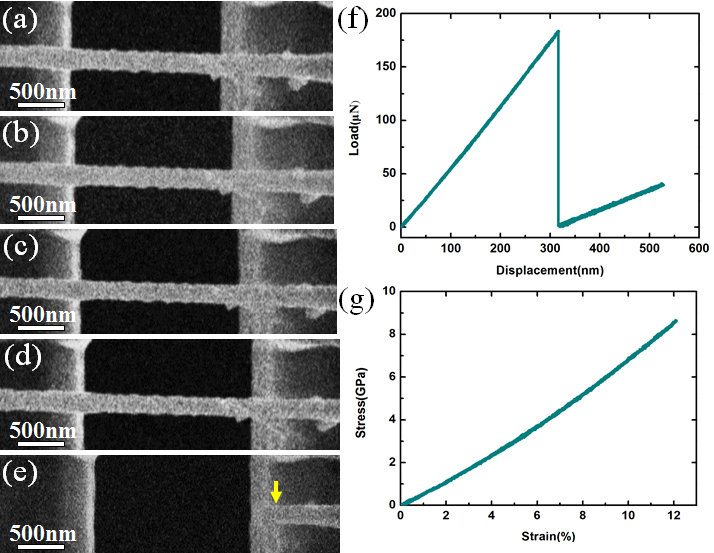


**Supplementary Figure S3.** In situ tensile test. (**a**)-(**e**) A sequence of snapshots taken from Movie S3 showing the tensile process of a single SiC NW with an ODD occupation ratio of 37.2%; (**f**) Force-Displacement curve of the single SiC NW; (**g**) Stress-strain curve of the single SiC NW.

1. MD simulation

The MD simulations are performed with using the large-scale atomic molecular massively parallel simulator (LAMMPS), and the vashishta potential ^R1^ is applied for the silicon and carbon atoms. ODD segments are simulated with a stacking sequence with a random order of ACABABCB…, ODD segments are distributed in the SiC NW evenly with a given occupation ratio, for example, the ratio in Figure 6 is 1:2. The model of the nanowire has a length of 45.279 nm, a width of 5 nm, and a thickness of 5 nm, contains about 112926 atoms. Periodic boundary condition is adopted along the nanowire axis. Before deformation, the model is relaxed for 50 ps with a NPT ensemble to ensure pressure in [11-1] direction approaches to nearly zero. The model is then loaded in a NVT ensemble until the fracture of the nanowire, which corresponds to an applied strain rate of 1ⅹ10^8^ s^-1^.

In order to study the twin lamellae effect, we constructed twin lamellae in ODD region and conducted the tensile test. Supplementary Fig. S4 shows the tensile test of a simulated NW with twin lamellae inserted into the ODD region, these two different shadow areas denoted ODD region and twin lamellae, respectively as shown in Supplementary Fig. S4a. Unlike Fig. 6, fracture happened in the twin lamellae region as shown in Supplementary Fig. S4c with a slight strength decrease comparing with the nanowire in Supplementary Fig. 6 with an ODD occupation ratio of 33.3% (Supplementary Fig.S4e).


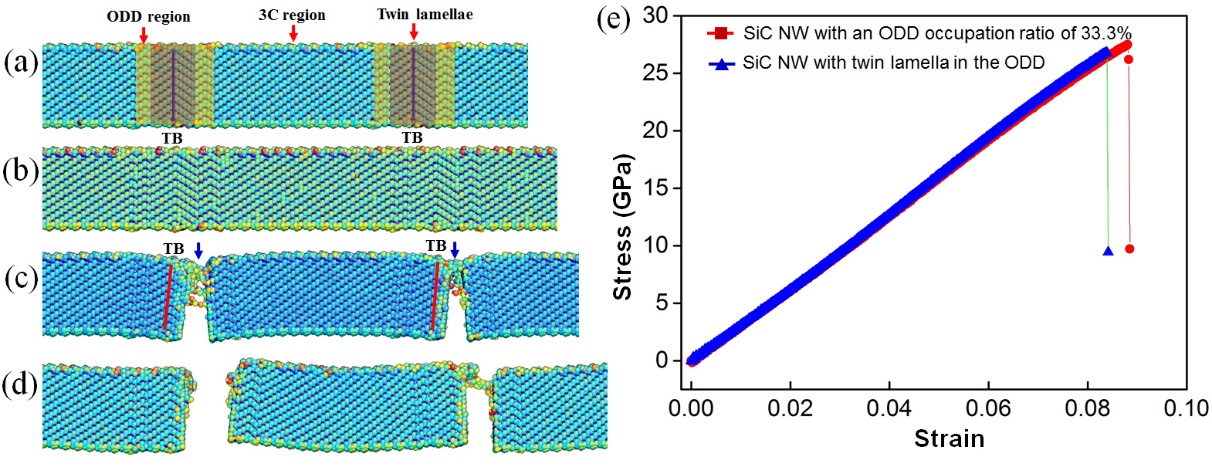


**Supplementary Figure S4.** MD simulation result of the SiC nanowire with periodical ODD structure, twin lamellae were inserted in the ODD region. (**a**)-(**d**) show the extracted snapshots taken from the tensile process; (**e**) Stress-strain curves of the SiC NW with different structures.

References:

R1. Vashishta, P., Kalia, R. K., Rino, J. P. & Ebbsjo, I. Interaction potential for: a molecular-dynamics study of structural correlations. *Phys. Rev. B.* **41,**12197 (1990).
